# Deregulated expression of TANK in glioblastomas triggers pro-tumorigenic ERK1/2 and AKT signaling pathways

**DOI:** 10.1038/oncsis.2013.42

**Published:** 2013-11-11

**Authors:** J Stellzig, A Chariot, K Shostak, S Ismail Göktuna, F Renner, T Acker, A Pagenstecher, M L Schmitz

**Affiliations:** 1 Institute of Biochemistry, Justus-Liebig-University, Medical Faculty, Friedrichstraße 24, Gießen, Germany; 2 Laboratory of Medical Chemistry, GIGA-Signal Transduction, University of Liège, C.H.U. Sart Tilman, Liège, Belgium; 3 WELBIO, University of Liège, C.H.U. Sart Tilman, Liège, Belgium; 4 Institute of Neuropathology, Justus-Liebig-University, Aulweg 123, Gießen, Germany; 5 Department of Neuropathology, University of Marburg, Baldingerstraße, Marburg, Germany

**Keywords:** glioblastoma, TANK, TBK1, NF-κB, inflammation, ERK

## Abstract

Signal transmission by the noncanonical IkappaB kinases (IKKs), TANK-binding kinase 1 (TBK1) and IKKɛ, requires interaction with adapter proteins such as TRAF associated NF-κB activator (TANK). Although increased expression or dysregulation of both kinases has been described for a variety of human cancers, this study shows that deregulated expression of the TANK protein is frequently occurring in glioblastomas (GBMs). The functional relevance of TANK was analyzed in a panel of GBM-derived cell lines and revealed that knockdown of TANK arrests cells in the S-phase and prohibits tumor cell migration. Deregulated TANK expression affects several signaling pathways controlling cell proliferation and the inflammatory response. Interference with stoichiometrically assembled signaling complexes by overexpression or silencing of TANK prevented constitutive interferon-regulatory factor 3 (IRF3) phosphorylation. Knockdown of TANK frequently prevents constitutive activation of extracellular signal-regulated kinases 1 and 2 (ERK1/2). TANK-mediated ERK1/2 activation is independent from the canonical MAP kinase or ERK kinase (MEK) 1/2-mediated pathway and utilizes an alternative pathway that uses a TBK1/IKKɛ/Akt signaling axis, thus identifying a novel pathway suitable to block constitutive ERK1/2 activity.

## Introduction

Glioblastoma multiforme (GBM) is the most common malignant primary brain tumor, which is characterized by morphological heterogeneity, as also reflected by its original description as ‘glioblastoma multiforme'.^1^ The histological and molecular diversity of GBMs creates a state where tumor growth is fueled by the simultaneous activity of multiple signaling pathways. The ability of GBMs to diffusely infiltrate throughout the brain tissue and its resistance to chemotherapy restricts median survival to only 14 months. Extensive profiling of GBM genomes and their epigenetic status has allowed the identification of at least four subtypes of GBM: classical, mesenchymal, proneural and neural.^2, 3^ Although each subtype is characterized by specific groups of frequently occurring mutations and deregulated signaling pathways, further mutations stereotypically occur in all GBM subgroups. These include mutations in epidermal growth factor receptor and further receptor tyrosine kinase pathways.^4^

Various mutations activate few oncogenic pathways that closely interact to produce an oncogenic signaling network that is instrumental in the expression and maintenance of a malignant phenotype. Aberrant activation of the MAP kinase or ERK kinase (MEK) 1/2 and extracellular signal-regulated kinases 1 and 2 (ERK1/2) occurs in many types of human cancers.^5^ Sustained ERK signaling is a requirement for S-phase entry and allows proliferation of tumor cells.^6^ GBMs often show constitutive activation of the pro-survival kinase AKT.^7^ The full activity of this kinase depends on Phosphoinositide-dependent protein kinase 1-mediated phosphorylation of T308 in the activation loop and mammalian target of rapamycin-triggered phosphorylation of S473 in the hydrophobic domain. These phosphorylations can be either triggered in a phosphatidylinositol 3 kinase-dependent manner or alternatively both sites can be directly phosphorylated by the noncanonical IkappaB kinases (IKKs), TANK-binding kinase 1 (TBK1) or IKKɛ.^8, 9, 10^ IKKɛ is also called inducible IκB kinase, as its mRNA expression can be augmented by lipopolysaccharide. IKKɛ and TBK1 are related to the canonical IKKs,^11^ which are key components of the classical nuclear factor-κB (NF-κB) activation pathway. Although the proinflammatory functions of IKKɛ and TBK1 in immune signaling rely on their ability to phosphorylate interferon-regulatory factor (IRF) 3 and 7 and NF-κB subunits, genetic studies have revealed that deregulated noncanonical IKKs are causative for metabolic diseases, oncogenesis and chemoresistance.^12, 13^ Point mutations are only rarely detected, but overexpression is frequently found in a variety of cancers including gliomas.^14^ Overexpression of IKKɛ is observed in ovarian and pancreatic cancer and most strikingly in one third of breast cancers where it has transforming activity. TBK1 has been linked to Ras-induced oncogenic transformation where activation of Ral-GEF leads to the formation of the exocyst complex consisting of TBK1, RalB and Sec5.^15^ Constitutive TBK1 activation supports oncogenic transformation and suppresses oncogene-induced apoptosis.^16, 17^ Both noncanonical IKKs are found in association with at least four adapter proteins called TRAF associated NF-κB activator (TANK), SINTBAD, optineurin and NAP1. Detailed biochemical studies showed mutual exclusive interaction of the adapters with their kinases, thus revealing the existence of distinct alternative protein complexes.^18, 19^ All four adapters compete for binding to a C-terminal coiled-coil region of TBK1, an interaction that is required for TBK1 function.^20, 21^

Adapter proteins typically lack intrinsic enzymatic activities, but act as platforms to assemble functional complexes.^22^ Therefore, adapter proteins coordinate signal specificity, aid to define the quality of the signal output and allow crosstalk with other signaling pathways. Accordingly, TANK is required to allow a physical and functional crosstalk between noncanonical and classical IKK complexes.^23, 24^ TANK does not function as a general regulator of NF-κB signaling and rather functions to restrict Toll-like receptor 7-triggered activation of NF-κB and AP1.^25, 26^

Here we show that GBMs frequently display extremely variable expression levels of the TANK protein. Knockdown experiments allowed the discovery of novel pro-proliferative signaling pathways, as TANK depletion frequently prohibited constitutive phosphorylation of ERK1/2, AKT and IRF3. Loss of TANK expression impaired S-phase progression and cell migration, thus revealing that also dysregulation of adapter proteins can affect tumorigenic signaling networks.

## Results

### Dysregulated TANK expression in GBMs

**Figure 1:**
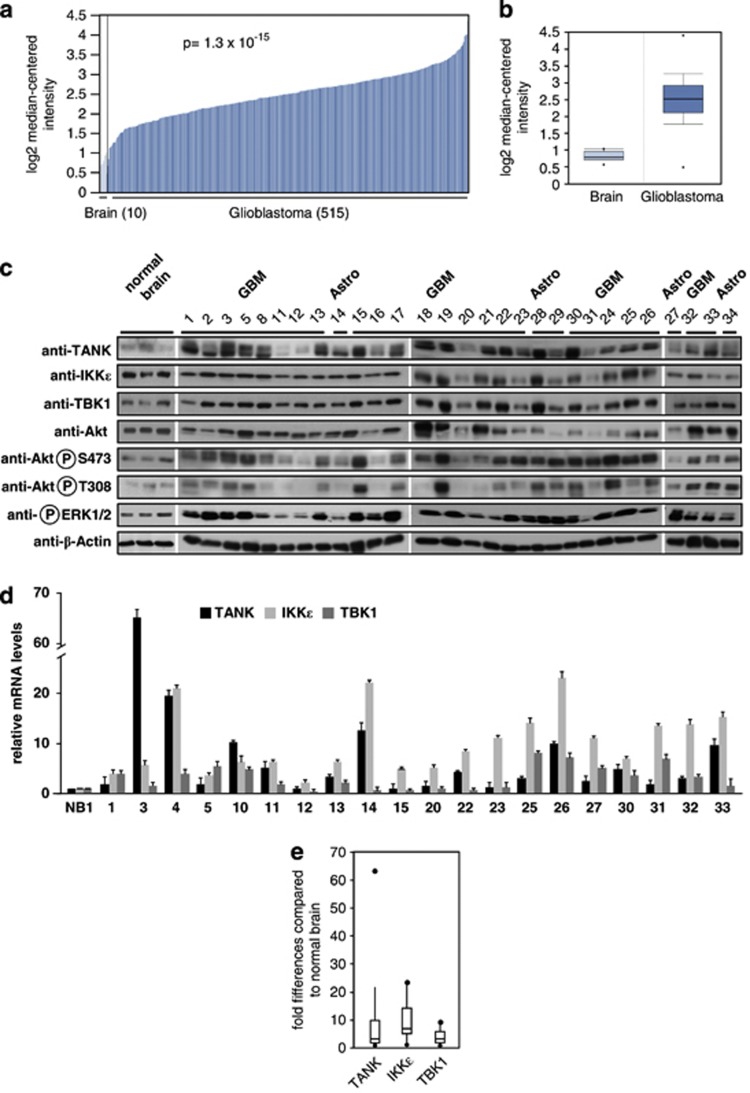
Deregulated expression of members of the noncanonical IKK complex in GBMs. (**a**) Analysis of TANK expression in normal brain (light blue) and GBMs (dark blue). Each bar represents an individual tissue sample (Reporter ID: 209451_at; Nucleotide Acc. No.: U59863). (**b**) Box plots of data from the Oncomine data sets comparing healthy brains and GBMs plotted on a log scale. Median values are shown by horizontal bars, the upper part of the box shows 75th percentile and the lower part the 25th percentile. The upper part of the bar shows 90th percentile and the lower part the 10th percentile. The points show outlier values, the figure was created using the Oncomine 3.0 software. (**c**) Comparable amounts of total proteins extracted from GBMs, astrocytomas and healthy brain were separated by sodium dodecyl sulfate–polyacrylamide gel electrophoresis, sample numbers are shown at the top. After blotting to a membrane, the samples were analyzed by immunoblotting for the occurrence and phosphorylation state of the indicated proteins with specific antibodies. The antibodies detecting β-actin were used to ensure comparable protein loading. As the samples could not be analyzed on one gel, the displayed exposure times were chosen to allow direct comparison between the samples. (**d**) RNA was extracted from the indicated samples where available. After preparation of complementary DNA, the relative expression amounts of TANK, IKKɛ and TBK1 were determined by quantitative PCR (qPCR) using specific primers. Expression in normal brain (NB) was arbitrarily set as 1, error bars show standard deviations from two experiments performed in triplicate. (**e**) Box plot analysis of the data shown in (**d**). Horizontal bars show median values, vertical bars show the standard deviations and the points show outlier values.

**Figure 2:**
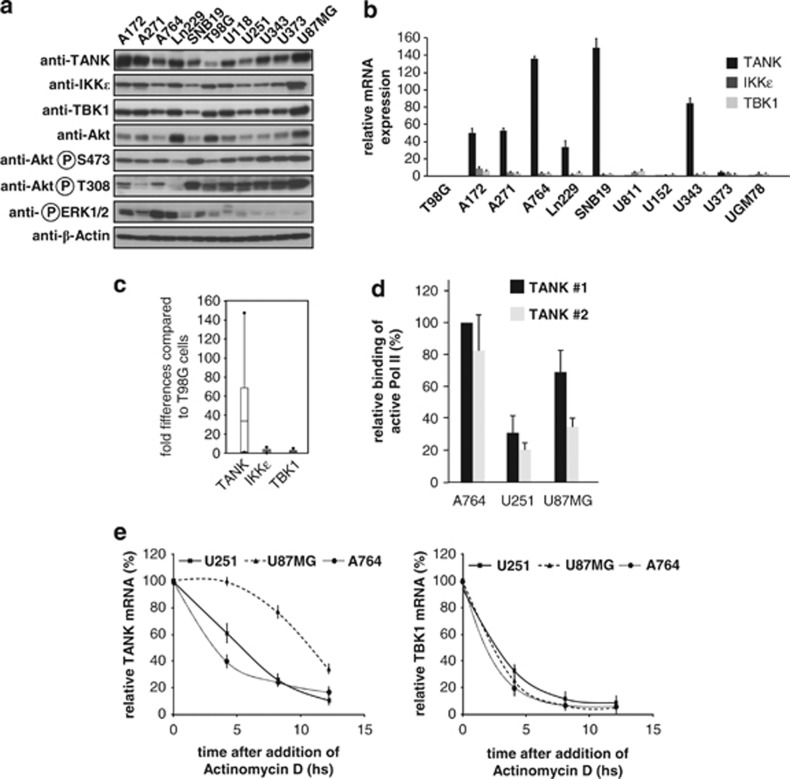
Deregulated expression of members of the noncanonical IKK complex in glioma cell lines. (**a**) Equal amounts of proteins extracted from the indicated cell lines were analyzed by immunoblotting for expression and phosphorylation of the indicated proteins. Exposure times were chosen in a way that allows comparison between cell lines in the dynamic range of the film. (**b**) Comparative analysis of mRNAs encoding TANK, IKKɛ and TBK1. The mRNA levels were quantified in the indicated cell lines by quantitative PCR (qPCR). Expression levels in T98G cells were arbitrarily set as 1, error bars are derived from two experiments performed in triplicates. (**c**) Box plot analysis of the data shown in (**b**). Vertical bars show the standard deviations, horizontal bars show median values and the points show outlier values. (**d**) Proteins were crosslinked to the DNA with formaldehyde in the indicated cell lines and chromatin immunoprecipitation (ChIP) assays were performed using a Pol II phospho S2 antibody or an unspecific IgG control antibody. Binding of Pol II S2p to the *Gapdh* control and two regions (#1 and #2) in the *Tank* gene were quantified by qPCR. To allow a direct comparison between the cell lines, data were normalized for binding of phosphorylated Pol II to the *Gapdh* gene. Maximal binding of phosphorylated Pol II was arbitrarily set as 100%, standard deviations are shown. (**e**) The different cells were treated with actinomycin D (1 μg/ml) for the indicated periods, followed by RNA isolation, complementary DNA production and the analysis of expression levels by qPCR. Error bars show standard deviations from two experiments performed in triplicates.

Given the elevated expression of IKKɛ in a variety of cancers including gliomas^13, 27^ and the involvement of TBK1 in oncogenesis, it was then interesting to investigate potential expression changes of the associated adapter protein TANK. To address this question, we performed an *in silico* expression analysis by comparing different microarray studies of various normal and tumor tissues from the Oncomine integrated cancer database (http://www.oncomine.org). The data sets revealed a strongly increased expression of TANK mRNAs in GBMs (Figures 1a and b). To investigate whether increased TANK levels are also seen at the protein level, western blotting experiments with samples from excised GBMs (WHO grade IV) and anaplastic astrocytomas (WHO grade III) were performed. These studies uncovered highly variable expression levels of TANK and also of the associated kinases TBK1 and IKKɛ (Figure 1c). In line with published literature,^28, 29^ also phosphorylation and thus activation of kinases allowing survival (AKT) and proliferation (ERK1/2) of tumor cells was frequently elevated in the gliomas (Figure 1c). The quantitative analysis of transcripts from the samples where RNA was also available showed that the highly dynamic range of protein expression levels is reflected at the mRNA level (Figures 1d and e). As the excised GBM material contains heterogeneous cell populations and also some nontransformed cells surrounding the tumor, it was then interesting to compare TANK expression levels in GBM cell lines. As each tumor cell line has specific defects in individual signaling networks, we analyzed a battery of different GBM and astrocytoma cell lines in order to get a more general picture. Protein expression levels of TANK showed a great variability with the relatively lowest levels in T98G cells and much less variability for IKKɛ and TBK1 (Figure 2a). Most cell lines also showed constitutive phosphorylation of AKT and ERK1/2 kinases. The analysis of mRNA expression revealed highly variable and elevated expression levels of TANK in 6 of the 11 analyzed cell lines (Figure 2b). Increased mRNA expression was restricted to TANK and not observed for TBK1/IKKɛ (Figure 2c). This might be due to the lack of tumor-associated cell populations creating a proinflammatory microenvironment that in turn could cause an elevated transcription of the kinases in cell lines. To investigate the mechanisms responsible for differential TANK expression, we compared cell lines characterized by high (A764) or low (U251 and U87MG) TANK transcript levels. *De novo* synthesis of TANK mRNA was measured using an indirect approach, as occupation of the TANK gene by active RNA polymerase II (marked by phosphorylation at S2) was used as an indicator of ongoing transcription. Chromatin immunoprecipitation experiments showed the increased occurrence of active RNA Pol II at the *Tank* gene in A764 cells (Figure 2d), thus revealing clear differences in ongoing transcription. To compare the mRNA stabilities between the cell lines, cells were treated for various periods with the transcription inhibitor actinomycin D, followed by determination of mRNA levels by quantitative PCR. These experiments showed significant variations in TANK mRNA stabilities, but largely unchanged levels of TBK1 transcripts (Figure 2e).

**Figure 3:**
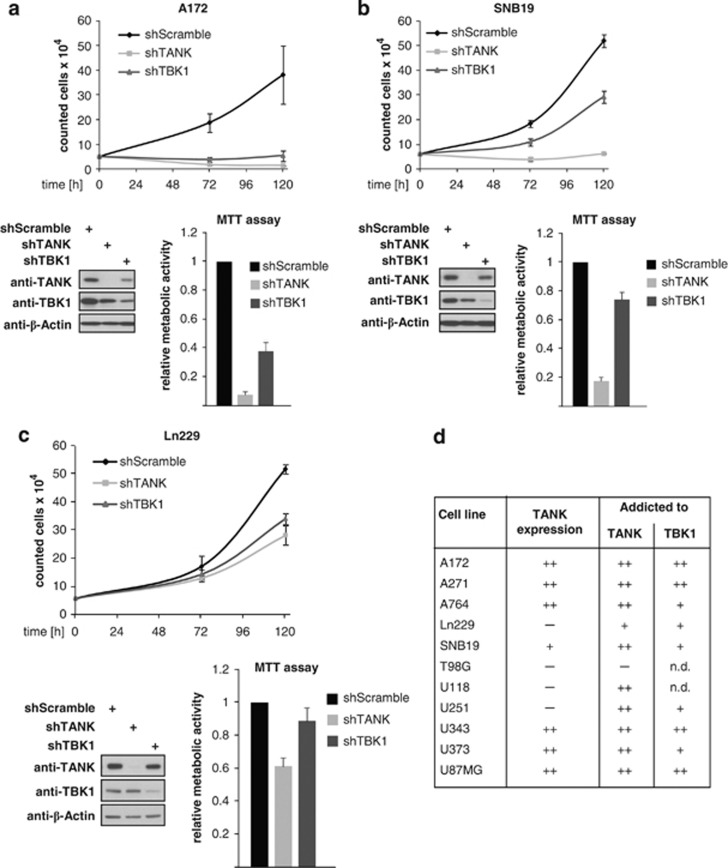
Many GBM cell lines are addicted to TANK. (**a**) A172 cells were retrovirally transduced to express short hairpin RNAs (shRNAs) leading to a knockdown of TANK, TBK1 or a scrambled shRNA as a control. Transduced cells were selected with puromycin and used to determine cell proliferation (upper) or alternatively protein expression and cell viability (lower). Proliferation was measured by plating out equal numbers of cells, followed by determination of cell numbers after 3 and 5 days using a FACSCalibur. Error bars show standard deviations from three different experiments. Aliquots of the cells were lysed and tested by western blotting for efficient knockdown. Equal numbers of cells were also seeded and used 2 days later for MTT assays, which reflect the metabolic activities and cell proliferation rates. Relative metabolic activity of control cells was arbitrarily set as 1, error bars show standard deviations. (**b** and **c**) The experiment was done as in (**a**) but it shows the experiment for cells, which are representative for groups that are only partially addicted from TBK1 (**b**) or TBK1 and TANK (**c**). (**d**) Summary of the cell proliferation assays. ++ shows completely absent and + shows impaired proliferation after knockdown. ND indicates not determinable, as TBK1 knockdown in T98G and U118 cells turned out not to be efficient.

Knockdown experiments were performed to test the functional relevance of TANK and its associated kinase TBK1 for proliferation of GBM cells. As exemplified for A172 cells, the proliferation of four cell lines was completely dependent on the expression of both proteins (Figure 3a). Of note, the knockdown of TANK also downregulated TBK1 and *vice versa*, reinforcing the notion that TBK1 and TANK stabilize each other to promote cell proliferation. Further four cell lines (as exemplified by SNB19 cells) did not grow after knockdown of TANK, whereas depletion of TBK1 only slightly retarded cell proliferation (Figure 3b). TANK knockdown had only moderate or even no inhibiting effect on the proliferation of Ln229 (Figure 3c) and T98G cells, respectively. These experiments are summarized in Figure 3d and collectively show a complete or at least partial dependence on TANK expression in 10 out of 11 cell lines. TBK1 critically drives cell proliferation in nine cell lines. However, its contribution could not be assessed in both T98G and U118 cells, as we failed to significantly deplete TBK1 in those cells (data not shown).

### TANK is required for S-phase transition and cell migration

**Figure 4:**
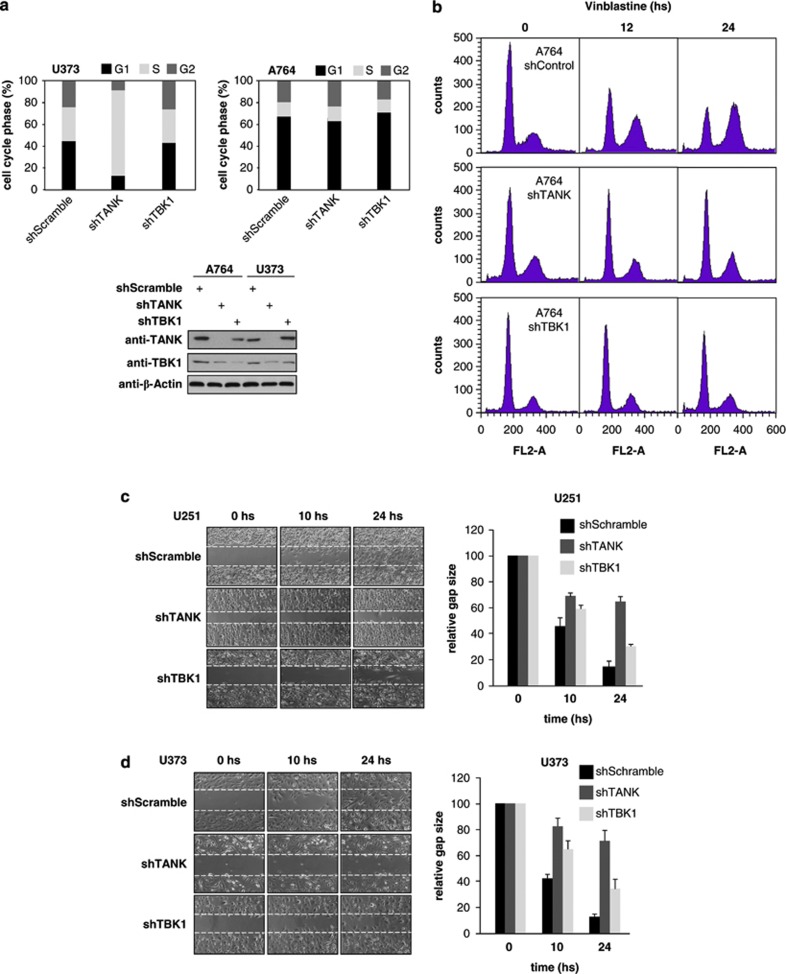
TANK can regulate the S-phase progression and contributes to cell migration. (**a**) The indicated cell lines were used to knockdown TANK or TBK1. After selection with puromycin, cells were used for quantitative analysis of cycle phases using a FACSCalibur (upper) and also for western blotting to ensure efficient knockdown (lower). (**b**) A764 cells were transduced to knock down expression of TANK or TBK1 as shown and then treated for the indicated periods with vinblastine. The cell cycle phases of propidium iodide stained cells were determined by fluorescence-activated cell sorting (FACS) as shown. (**c**) The indicated knockdown and control U251 cells were grown to density and treated with the DNA polymerase inhibitor aphidicolin (1 μg/ml) to prevent proliferation. After scratching of the cell monolayer migration of cells was examined using a NIKON Eclipse TE2000-E life cell imaging microscope (Nikon, Düsseldorf, Germany). The right part shows a quantitative analysis of cell migration, error bars were derived from two independent experiments performed in triplicates. (**d**) The experiment was done as in (**c**) with the exception that U373 cells were analyzed.

As TANK knockdown did not result in apoptosis (data not shown), it was then interesting to determine its impact on the cell cycle. In some cell lines, downregulation of TANK caused accumulation in S-phase, whereas other cell lines such as A764 cells did not show any TANK or TBK1-dependent changes of cell cycle phases (Figure 4a). To monitor the dynamic distribution of the cell cycle phases in A764 cells, control and knockdown cells were arrested at late G2 by treatment with vinblastine. One day after the addition of vinblastine, most of the control cells had accumulated at G2, whereas cells depleted for TANK or TBK1 remained almost entirely in the G1 or S-phase (Figure 4b), indicating that they cannot further progress throughout the cell cycle. As GBMs are characterized by their ability to migrate and diffusely infiltrate throughout the brain tissue,^4^ we then tested a possible contribution of TANK for cell migration. Scratch assays showed an almost complete closure of the gap after 24 h in control cells, whereas the downregulation of TANK strongly decreased the migration of GBM cells, as exemplified for U251 (Figure 4c) and U373 (Figure 4d) cells. In each case, the migration-restricting ability of TBK1 was less pronounced. Of note, depletion of TANK did not always result in inhibition of migration, as exemplified for T98G cells, which migrate even in the absence of TANK (Supplementary Figure S1).

### TANK and TBK1 expression promotes phosphorylation of Akt and ERK

**Figure 5:**
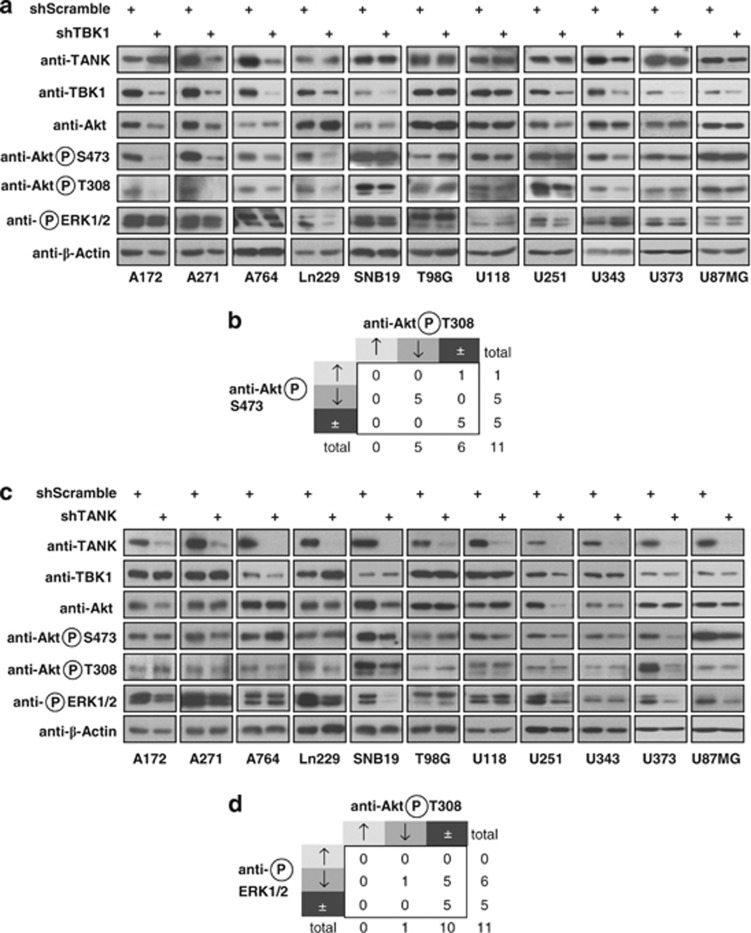
Identification of signaling pathways regulated by the noncanonical IKK complex. (**a**) The indicated cell lines were used to knock down TBK1 and subsequently analyzed for protein expression and phosphorylation with the indicated antibodies. (**b**) The TBK1-dependent coregulation between AKT S473 and T308 phosphorylation is schematically displayed, downward arrows show inhibition of phosphorylation. (**c**) The experiment was done as in (**a**) with the exception that TANK was knocked down. (**d**) Schematic display of absent coregulation between AKT and ERK1/2 phosphorylation after downregulation of TANK.

To further understand how TBK1 and TANK promote cell proliferation and migration at the molecular level in GBM-derived cells, cells were depleted for each of these proteins and then analyzed for the activity status of AKT and ERK1/2 with phospho-specific antibodies. Knockdown of TBK1 impaired the constitutive AKT phosphorylation at T308 and S473 in five cell lines (A172, A271, A746, Ln229 and U343; Figures 5a and b and Supplementary Figure S2), showing its important contribution that cannot be compensated by Phosphoinositide-dependent protein kinase 1-derived or mammalian target of rapamycin-derived phosphorylations. Interestingly, TBK1 knockdown resulted in a slight and partial impairment of constitutive ERK1/2 phosphorylations in four cell lines (A271, Ln229, SNB19 and U251). Downregulation of TANK impaired AKT phosphorylation only in U373 and U251 cells, whereas it strongly precluded ERK1/2 phosphorylation in six cell lines (A172, A271, Ln229, SNB19, U251 and U373; Figures 5c and d and Supplementary Figure S3).

**Figure 6:**
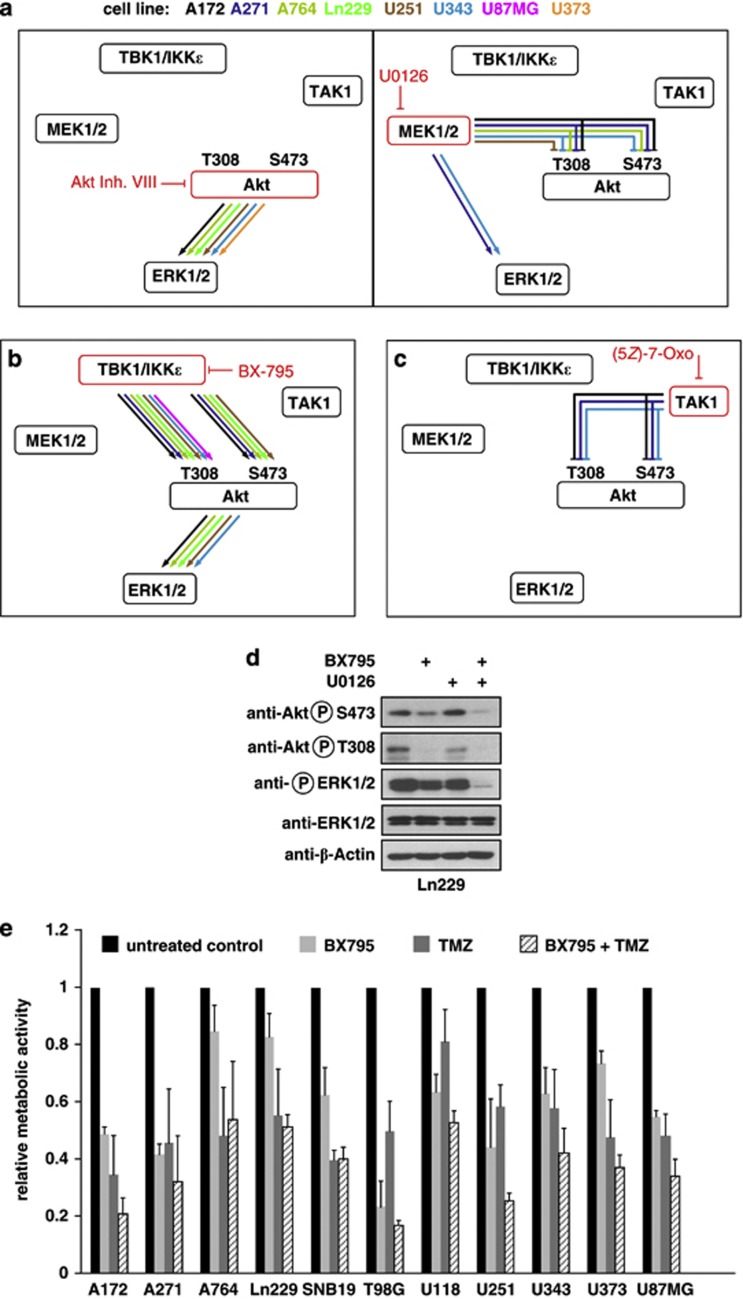
Noncanonical IKKs activate ERK1/2 via AKT. (**a**) The indicated cell lines were incubated overnight with the AKT inhibitor VIII (5 μM) or with the MEK1/2 inhibitor U0126 (5 μM), followed by analysis of AKT phosphorylation at T308 or S473 and ERK1/2 phosphorylation by immunoblotting. The results are schematically displayed, the inhibited enzymes are shown in red, the necessity of enzyme activity (as revealed by significantly impaired or absent phosphorylation) is shown by arrows representing the different cell lines. Inhibitory kinase activities (as revealed by exaggerated phosphorylations in the presence of kinase inhibitors) are shown by lines lacking an arrow head. (**b**) Impact of BX795-mediated TBK1/IKKɛ inhibition and (**c**) 7-oxozeaenol-mediated TAK1 inhibition on the signaling networks. The original blots for the data sets schematically summarized in (**a**–**c**) are shown in Supplementary Figure S8. (**d**) Ln229 cells were incubated with BX795 (1 μM) and U0126 (5 μM) at the indicated combinations and phosphorylation of ERK1/2 was analyzed by immunoblotting. (**e**) The indicated GBM cell lines were incubated for 72 h with BX795 (1 μM), temozolomide (TMZ; 100 μM) or a combination of both. Cell viability was determined by MTT assays, error bars show standard deviations from two experiments performed in triplicate.

To investigate the signaling networks delivering TANK-derived signals to ERK1/2 in TANK addicted cells, phosphorylations were monitored after inhibition of specific kinases. ERK1/2 phosphorylation can be mediated either by the upstream kinases MEK1/2^30^ or alternatively by AKT-derived signals.^31^ To test the relative contribution of these two pathways, cells were grown in the presence of the MEK1/2 inhibitor U0126 or the Akt inhibitor VIII. The analysis of ERK1/2 phosphorylation showed that the vast majority of GBM cell lines rely on AKT-delivered signals (Figure 6a). Of note, inhibition of MEK1/2 often resulted in exaggerated AKT phosphorylation, indicating the possible existence of a negative feedback loop. Inhibition of the TANK-associated kinases TBK1/IKKɛ with BX795 resulted in absent phosphorylation of AKT and ERK1/2 in the majority of cell lines, showing that these kinases lead to AKT-dependent ERK1/2 activation (Figure 6b). Inhibition of TAK1, a kinase with a central role for NF-κB signaling,^32^ remained without impact on ERK1/2 but resulted in enhanced AKT phosphorylation (Figure 6c), probably due to another negative feedback loop. Major efforts have been undertaken to inhibit constitutive activity of ERK1/2 in cancer,^33^ but cancer cells are frequently resistant to MEK1/2 inhibitors.^34, 35^ As we identified a novel TANK-AKT signaling pathway leading to ERK1/2 activation, we tested whether the combined inhibition of MEK1/2 and TBK1/IKKɛ-derived signals could efficiently turn off ERK1/2 activation. As shown in Figure 6d, treatment with BX795 partially inhibited ERK1/2 activities, whereas the combination of BX795 with U0126 resulted in a complete blockage of ERK1/2 phosphorylation.

GBMs are frequently treated with the chemotherapeutic agents temozolomide or cytarabine. To investigate whether the therapeutic efficacy of these drugs can improve upon concomitant TBK1/IKKɛ inhibitions, temozolomide was administered either alone or in combination with BX795 to GBM cell lines. Temozolomide-induced cell death was enhanced upon simultaneous inhibition of TBK1/IKKɛ signaling, but beneficial effects were only moderate (Figure 6e) or even absent when BX795 and cytarabine treatments were combined (Supplementary Figure S4). These findings are consistent with the concept that chemotherapeutic drugs only affect rapidly proliferating cells and not cells that are slowed down in their proliferation upon interference with noncanonical IKK signaling.

### TANK regulates inflammatory signaling cascades

**Figure 7:**
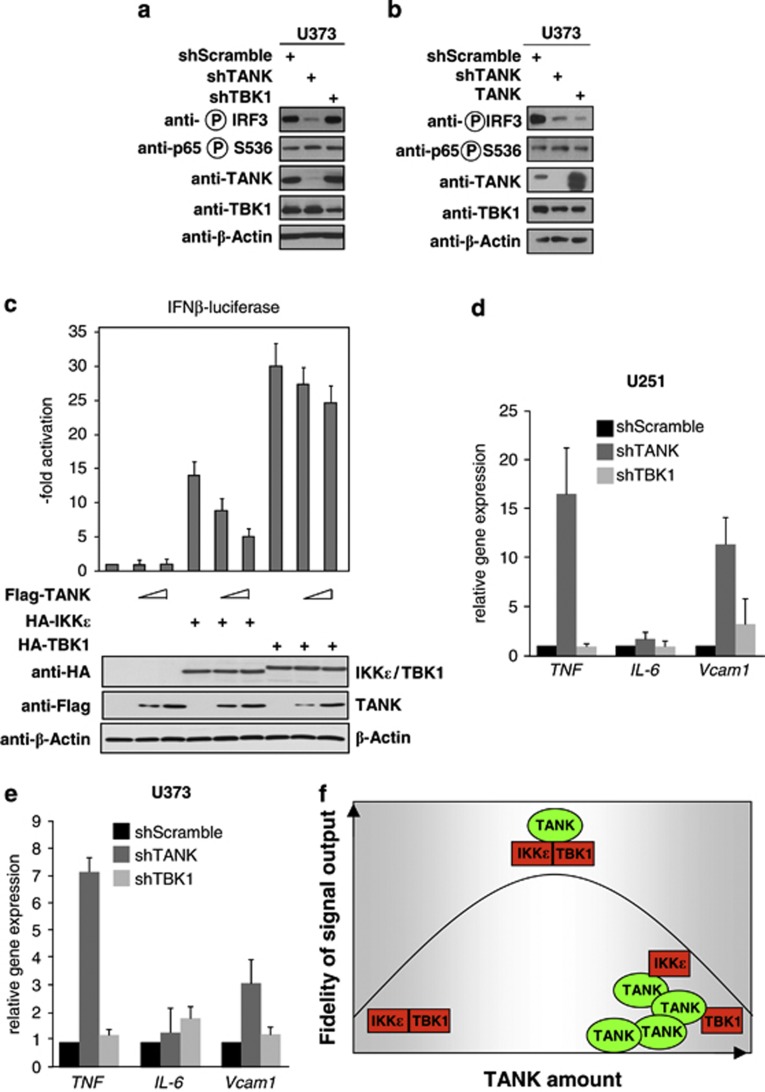
Functional consequences of deregulated TANK expression. (**a**) TANK or TBK1 were knocked down in U373 cells with specific short hairpin RNAs (shRNAs), followed by the analysis of NF-κB p65 and IRF3 phosphorylation with specific antibodies. (**b**) TANK was overexpressed in U373 cells and extracts were analyzed for NF-κB p65 and IRF3 phosphorylation by western blotting. (**c**) HEK293 cells were transfected with a plasmid encoding the luciferase gene under the control of the interferon β (IFNβ) promoter along with the indicated expression vectors. After 36 h, cells were lysed and one aliquot was used for the determination of luciferase activity (upper) and the other aliquot was used to determine adequate protein expression. Error bars were derived from two experiments performed in triplicate. (**d** and **e**) TANK or TBK1 were knocked down in U373 (**d**) or U251 (**e**) cells, followed by quantitative analysis of proinflammatory gene expression by quantitative PCR (qPCR). (**f**) Schematic model illustrating that the fidelity of signal output is critically determined by the amount of TANK and the stoichiometric assembly of functional signaling complexes.

As TANK physiologically dampens inflammatory signaling upon interference with TRAF6 activation,^25^ it was then interesting to investigate the contribution of TANK to proinflammatory signaling. Knockdown of TANK in U373 cells resulted in strongly impaired constitutive phosphorylation of IRF3 (Figure 7a), a transcription factor that may interfere with glioma proliferation, migration and invasion.^36^ Consistent with the concept that overexpression of adapter proteins interferes with signaling by preventing stoichiometric assembly of multi-protein signaling complexes,^37^ also overexpression of TANK resulted in the inhibition of IRF3 phosphorylation (Figure 7b). Accordingly, TBK1- or IKKɛ-triggered transcription of a luciferase reporter gene controlled by the interferon β promoter was impaired after TANK expression in a dose-dependent manner (Figure 7c). At the level of gene expression, downregulation of TANK resulted in elevated expression of the proinflammatory *Tnf* and *Vcam* genes, as revealed by quantitative PCR (Figures 7d and e). In contrast, knockdown of TBK1 remained without a major impact on the expression of these genes in unstimulated cells. These results suggest that deregulated TANK expression in tumors has not only consequences for cell proliferation but also on the signaling network mediating proinflammatory gene expression.

## Discussion

### Mechanisms of deregulated TANK expression in GBMs

Here we show that TANK overexpression and deregulated expression of noncanonical IKKs are frequently found in GBMs. The molecular mechanisms leading to elevated TANK expression remain unknown. Increased mRNA expression of the *Tank* gene may be due to overexpression of its positive regulator Sox11, a transcription factor that is frequently found to be overexpressed in gliomas.^38^ RNA expression levels as detected by gene array or RNA-seq experiments do not always correlate with protein expression levels, as exemplified by the U87MG cell line. Here, the relatively moderate mRNA expression levels are contrasted by robust expression of the TANK protein. As protein expression levels can be regulated by multiple mechanisms that include checkpoints controlling mRNA processing and stability, translation initiation as well as protein stability,^39^ mRNA levels are not necessarily reflecting protein abundance. As immune-regulating proteins controlling IRF and NF-κB signaling are frequently found to be regulated at each stage of gene expression,^40^ we rather speculate that elevated TANK expression is due to multiple mechanisms that affect mRNA synthesis and stability. Our data indicate differential stabilities of TANK mRNAs (Figure 2e) and accordingly the analysis of TANK mRNA revealed the occurrence of several distinct AU-rich elements that could potentially allow docking of RNA-binding proteins and regulation of mRNA stabilities (Supplementary Figure S5). Furthermore, a previous study described a specific recurrent chromosomal break region at the chromosome 2q24–2q32 region encompassing the TANK-encoding locus,^41^ extensive characterizations of GBM genomes failed to identify frequent gene rearrangements in the TANK-containing region.^42^ GBMs often show hypoxic areas with low availability of oxygen,^43^ raising the possibility that this could contribute to the regulation of TANK abundance. However, we failed to detect major changes in the mRNA and protein expression of TANK and noncanonical IKKs in hypoxic cells (Supplementary Figures S6A and B).

### TANK-regulated signaling pathways

Only optimal amounts of adapter proteins ensure correct signal output, as underexpression as well as overexpression cause faulty signal output, as schematically shown in Figure 7f. In that sense, the overexpressed TANK protein could compete with SINTBAD, NAP1 and optineurin for the association with the other >30 regulatory proteins that are known to interact with these adapters.^18^ This in turn can affect multiple signaling pathways simultaneously and thus cause a re-wiring and re-programming of signaling networks. Obvious candidate enzymes affected by deregulated TANK expression are the associated kinases TBK1 and IKKɛ and accordingly overexpression of IKKɛ has been associated with impaired apoptosis of glioma cells.^27^ Interestingly, an Oncomine database search for genes that are coregulated with TANK in brain tumors identified STAM2 (signal transducing adaptor molecule) as the top ranking hit (Supplementary Figure S7), a protein that is also known to function as an adapter protein.^44^ Besides the described effects of IKKɛ on cell survival, the noncanonical IKKs also mediate ERK1/2 activation, as concluded from the inhibitory activity of BX795 on constitutive ERK1/2 activity. The results from knockdown experiments and inhibitor studies suggest that TANK-derived signals travel via TBK1/IKKɛ to AKT, which then in turn allows ERK1/2 activation. It is currently unclear whether TBK1/IKKɛ directly activates ERK1/2, or whether it uses AKT as a connecting kinase. AKT-mediated ERK1/2 activation may rely on its reported ability to inhibit c-Jun N-terminal kinase, which in turn alleviates the repressive function of c-Jun N-terminal kinase on ERK1/2 signaling.^31^ As many tumor cells have learned to induce ERK1/2 phosphorylation by a MEK1/2-independent signaling route,^35^ the detailed characterization of the molecular mechanisms allowing for this bypass will be highly relevant. TANK-derived signals may not only travel via TBK1/IKKɛ, as ERK1/2 phosphorylation in A271 and U373 cells is affected by downregulation of TANK but not by BX795. This finding could be explained by an influence of TANK on other signaling molecules. One candidate is TRAF6 as a previous study showed the inhibitory activity of TANK on its function,^25^ but also any of the further signal transducing enzymes contained in the TANK/TBK1 molecular network may be responsible for these effects.^18^

This study also shows an effect of TANK on constitutive STAT3 phosphorylation, a transcription factor that mediates upregulation of interleukin-6 (IL-6), which in turn facilitates glioma development by promoting angiogenesis and cell proliferation.^45^ The impact of TANK on this signaling pathway may well be indirect, as TANK is known to contribute to a primary IFN response,^46^ which then triggers a secondary response involving STAT3 phosphorylation.^47^ However, TANK could also be involved in an alternatively STAT3 activating pathway downstream from the tyrosine kinase BMX.^48^

TANK also binds to NF-κB essential modulator, which is the adapter protein for the canonical NF-κB activating kinases IKKα and IKKβ.^24^ This interaction allows a crosstalk between TANK and classical NF-κB activity^23^ and thus different TANK levels may well affect the thresholds and kinetics of NF-κB signaling in GBMs. The functional role of NF-κB activation in GBMs is not yet clear and its aberrant activation may not be a cause but rather a consequence of the tumor microenvironment that is characterized by NF-κB activating conditions such as dysregulated cytokine expression and hypoxia.^49^

### Inflammation in GBM

The signaling pathways discussed above will lead to altered secretion of cytokines or growth regulatory factors, which then act in a paracrine or juxtacrine manner to influence cell proliferation. The role of inflammatory processes for the proliferation of GBMs does not lead to a coherent picture. Experiments with tumor cell lines show p38 mitogen-activated protein kinase-dependent expression of cytokines such as IL-1β, IL-6 and IL-8, which in turn trigger invasiveness of U251 GBM cells.^50^ Complex cytokine networks also mediate the interaction between tumor cells and untransformed cells in the GBM microenvironment, thus rendering tumor heterogeneity as an active process.^51^ On the other hand, cytokine expression levels occurring in tumor cells or normal brain cells do not correlate with histopathological parameters such as necrosis or ischemic necrosis.^52^ GBMs are also well known for cancer-associated immune inhibition by soluble factors such as transforming growth factor-β.^53^ Accordingly, immunosuppressive factors secreted by gliomas frequently result in a broad suppression of cell-mediated immunity.^54^

In summary, our results show that the deregulated expression of non-enzymatic adapter proteins such as TANK can have a profound influence on signal output from several signaling networks. Although adapter proteins are not druggable, interference with adapter-associated enzymes such as TBK1/IKKɛ can be useful to restrict aberrant bypassing of signaling pathways in tumor cells.

## Materials and methods

### Antibodies, plasmids and reagents

This information is given in the Supplementary Table S1.

### Chromatin immunoprecipitation

Proteins and DNA were crosslinked by incubation for 10 min with 1% (v/v) formaldehyde at room temperature, followed by quenching of formaldehyde with 0.125 M glycine for 2 min and lysis in RIPA buffer as described.^55^ A Branson sonifier 250 (Emerson, Danbury, CT, USA) was used to shear the genomic DNA and cellular debris was removed by centrifugation. RNAs were digested by incubation with RNase A, equal amounts of DNA were incubated with 2 μg of antibodies previously bound to protein G-coupled Dynabeads. After extensive washing, the precipitated DNA fragments were eluted. The sequences of the TANK-specific primers used for chromatin immunoprecipitation experiments are given in the supplementary section. The PCR product was quantified by real-time quantitative PCR with specific primers using the Applied Biosystems (Life Technologies, Carlsbad, CA, USA) 7300 real-time PCR system.

### Cell lines

HEK293 cells, 293T Phoenix Ampho packaging cells and all GBM cell lines (A172, A271, A764, G55, Ln229, SNB19, T98G, U118, U251 and U343) and astrocytoma lines (U373 and U87MG) were grown in Dulbecco's modified Eagle's medium supplemented with 10 % (v/v) fetal calf serum, 2 mM L-glutamine and 1 % (v/v) penicillin/streptomycin.

### Cell proliferation and MTT assays

Cells were trypsinized and counted by fluorescence-activated cell sorting analysis. Constant numbers of cells were plated on 6 cm dishes and further grown for 72 and 120 h. At these time points, cells were harvested by trypsinization and cell numbers were determined by fluorescence-activated cell sorting. Cell viability was quantified using the Vybrant MTT Cell Proliferation Assay Kit (Life Technologies, Carlsbad, CA, USA). Cells were seeded in a 96-well-plate at a density of 1 × 10^4^ cells per well. After 24 h, the cells were subjected to the indicated treatments and 1 day later cell viability was measured by adding 10 μl of the MTT stock solution (12 mM) to each well, followed by incubation at 37 °C for 4 h. To solubilize the formazan crystals formed, 100 μl of sodium dodecyl sulfate–HCl solution were added and the plate was incubated at 37 °C for 4–18 h in a humidified chamber. The viability was determined by measuring the absorbance at 570 nm.

### Production of retroviruses and lentiviruses

293T Phoenix Ampho packaging cells capable of producing gag-pol and envelope protein for amphotropic viruses were transfected with either pSIREN-Scramble, pSIREN-shTANK or pSIREN-shTBK1 vectors using Rotifect (Roth, Karlsruhe, Germany) according to the manufacturer's instructions. Two days after transfection of the virus-producing cells, the virus-containing supernatant was collected and filtered through a 0.45 μm filter. After adding polybrene to a final concentration of 5 μg/ml, the supernatant was added to the GBM cell lines for 24 h. Medium was subsequently changed back to culture medium. Three days post infection, GBM cell lines were treated with 2 μg/ml puromycin for at least 5 days to select short hairpin RNA-expressing cells. All key experiments were repeated with a second independent short hairpin RNA to exclude off-target effects. TANK was overexpressed using a lentiviral vector. Viruses were produced in HEK293T cells by transfecting a lentiviral TANK expression vector together with the packaging vectors pMDLg/pRRE, pRSV-Rev and pHCMVG using lipofectamine according to the manufacturer's instructions. Two days after transfection, the virus-containing supernatant was collected and used for infection of GBM cell lines.
