# Supplementary material for: Deregulated expression of TANK in glioblastomas triggers pro-tumorigenic ERK1/2 and AKT signaling pathways
Source: Oncogenesis. 2013 Nov 11;2(11):e79–. doi: 10.1038/oncsis.2013.42 (PMC3849693; doi:10.1038/oncsis.2013.42)
Supplement: Supplementary Figures [file oncsis201342x1.pdf]

Supplementary Figures

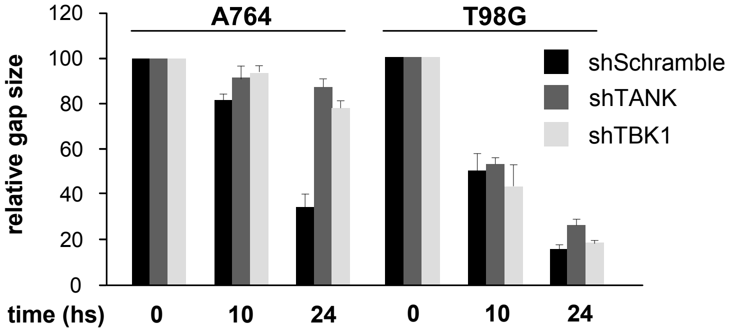

Figure S1. The indicated knockdown and control cells were grown to density and treated with the DNA polymerase inhibitor aphidicolin (1 μg/ml) to prevent proliferation. After scratching of the cell monolayer migration of cells was examined using a NIKON Eclipse TE2000-E life cell imaging microscope. The right part shows a quantitative analysis of cell migration, error bars were derived from 2 independent experiments performed in triplicates.

Silencing TBK1

anti-Akt(P) T308 (columns) vs. **anti-(P) ERK1/2** (rows)

	↑	↓	±	total
↑	0	0	0	0
↓	0	2	2	4
±	0	3	4	7
total	0	5	6	11

anti-Akt(P) S473 (columns) vs. **anti-(P) ERK1/2** (rows)

	↑	↓	±	total
↑	0	0	0	0
↓	0	2	2	4
±	1	3	3	7
total	1	5	5	11

Figure S2. GBM cell lines were used to knock down TBK1 and subsequently analyzed for protein expression and phosphorylation of ERK and Akt with specific antibodies. Coregulation between Akt T308 and ERK (left) as well as Akt S473 and ERK (right) is schematically shown.

Silencing TANK

anti-Akt(P) T308 (columns) vs. **anti-Akt(P) S473** (rows)

	↑	↓	±	total
↑	0	0	0	0
↓	0	1	1	2
±	0	0	9	9
total	0	1	10	11

anti-Akt(P) S473 (columns) vs. **anti-(P) ERK1/2** (rows)

	↑	↓	±	total
↑	0	0	0	0
↓	0	2	4	6
±	0	0	5	5
total	0	2	9	11

Figure S3. GBM cell lines were used to knock down TANK and subsequently analyzed for protein expression and phosphorylation of ERK and Akt with specific antibodies. Coregulation between Akt T308 and ERK (left) as well as Akt S473 and ERK (right) is schematically shown.

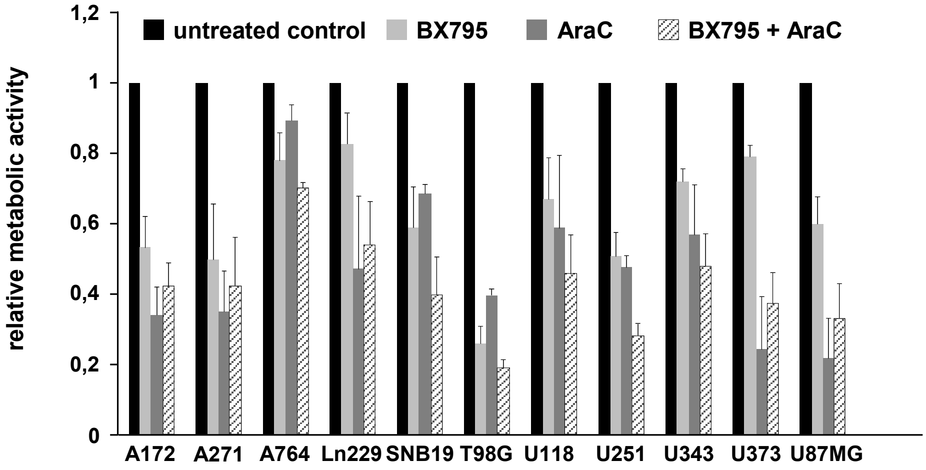

Figure S4. The indicated GBM cell lines were incubated for 72 hs with BX795 (1 μM), AraC (1 μM) or a combination of both. Cell viability was determined in a MTT assay, error bars show standard deviations from two experiments performed in triplicate.

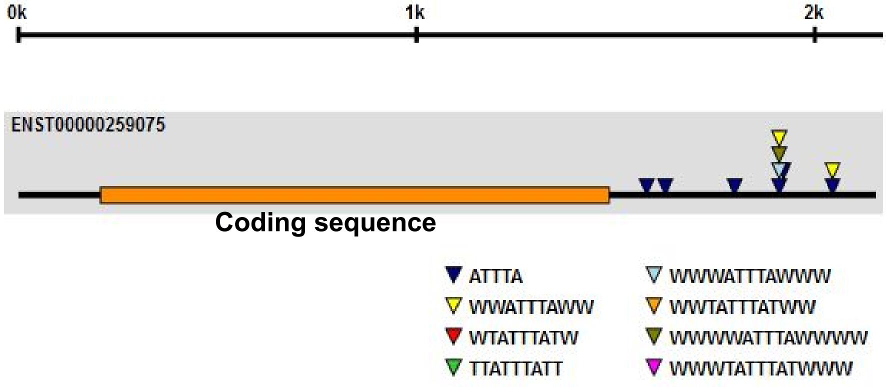

Figure S5. The TANK mRNA was analyzed for the occurrence of AU-rich elements (AREs) using the AREsite database (http://rna.tbi.univie.ac.at/AREsite). The TANK mRNA is schematically shown, the distribution of the various ARE sequences in the 3´ UTR is displayed. W stands for weak (A or T).

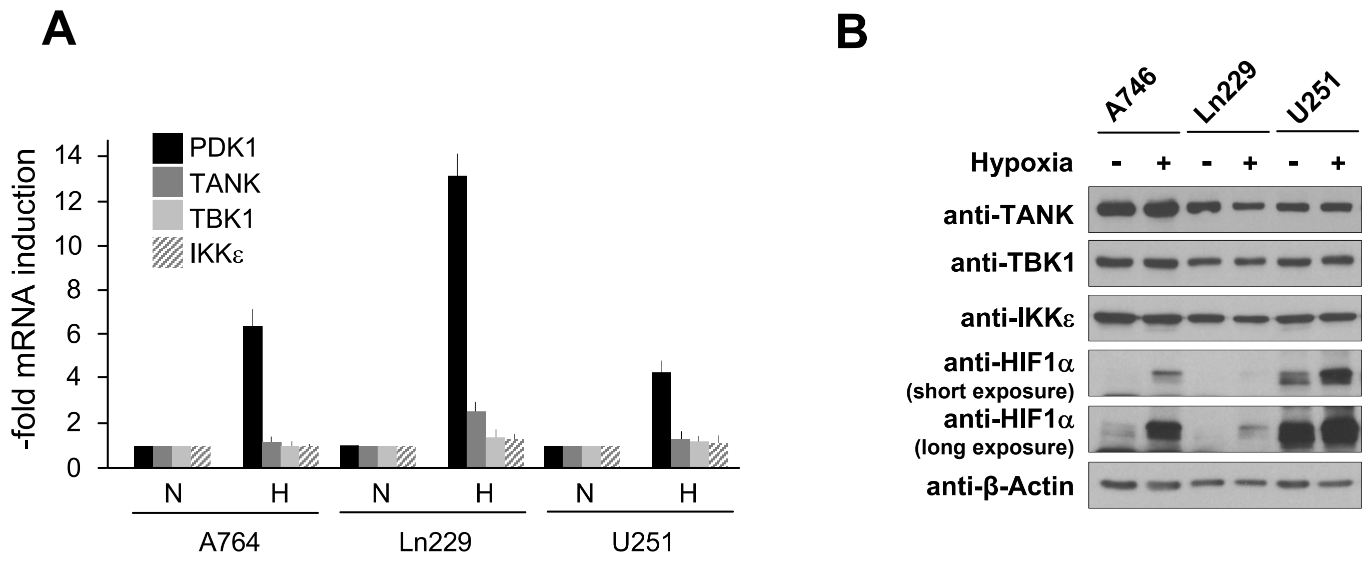

Figure S6. Effect of hypoxia (1% O_2) on expression of TANK and the noncanonical IKKs. **(A)** The indicated cells were incubated for 14 hs under normoxic (N) or hypoxic (H) conditions and mRNA of the indicated genes was quantified by qPCR. Pyruvate dehydrogenase kinase-1 (PDK1) was used as a positive control, error bars show standard deviations derived from two experiments performed in triplicate. **(B)** The cells were incubated for 14 hs under normoxic (N) or hypoxic (H) conditions and protein extracts were analyzed by Western blotting for the expression of the indicated proteins, hypoxia-inducible factor 1 α (HIF1α) was used as a positive control.

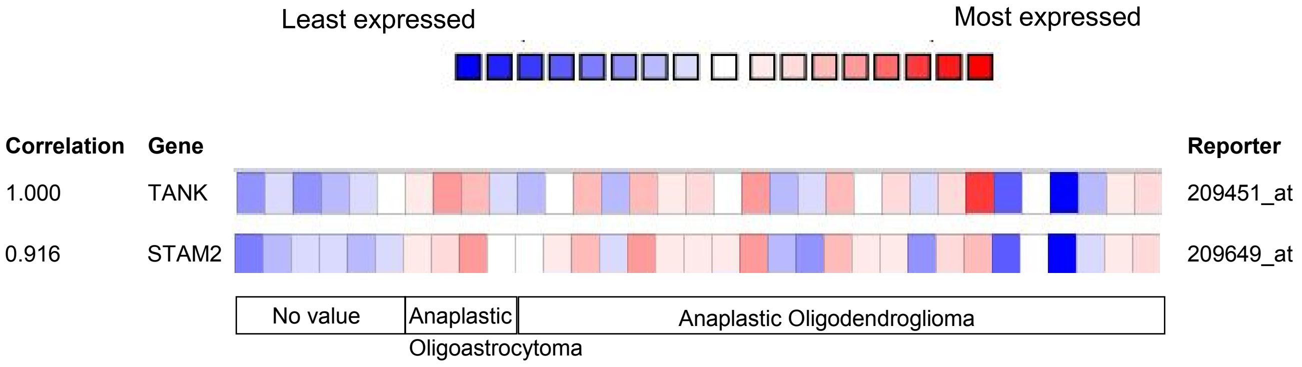

Figure S7. Coexpression analysis of TANK in gliomas. The coexpression filter in the Oncomine database was used to identify transcripts correlating with TANK expression. The top ranking gene was STAM2 with a correlation value of 0.916. Different expression levels are visualized by colors as shown.

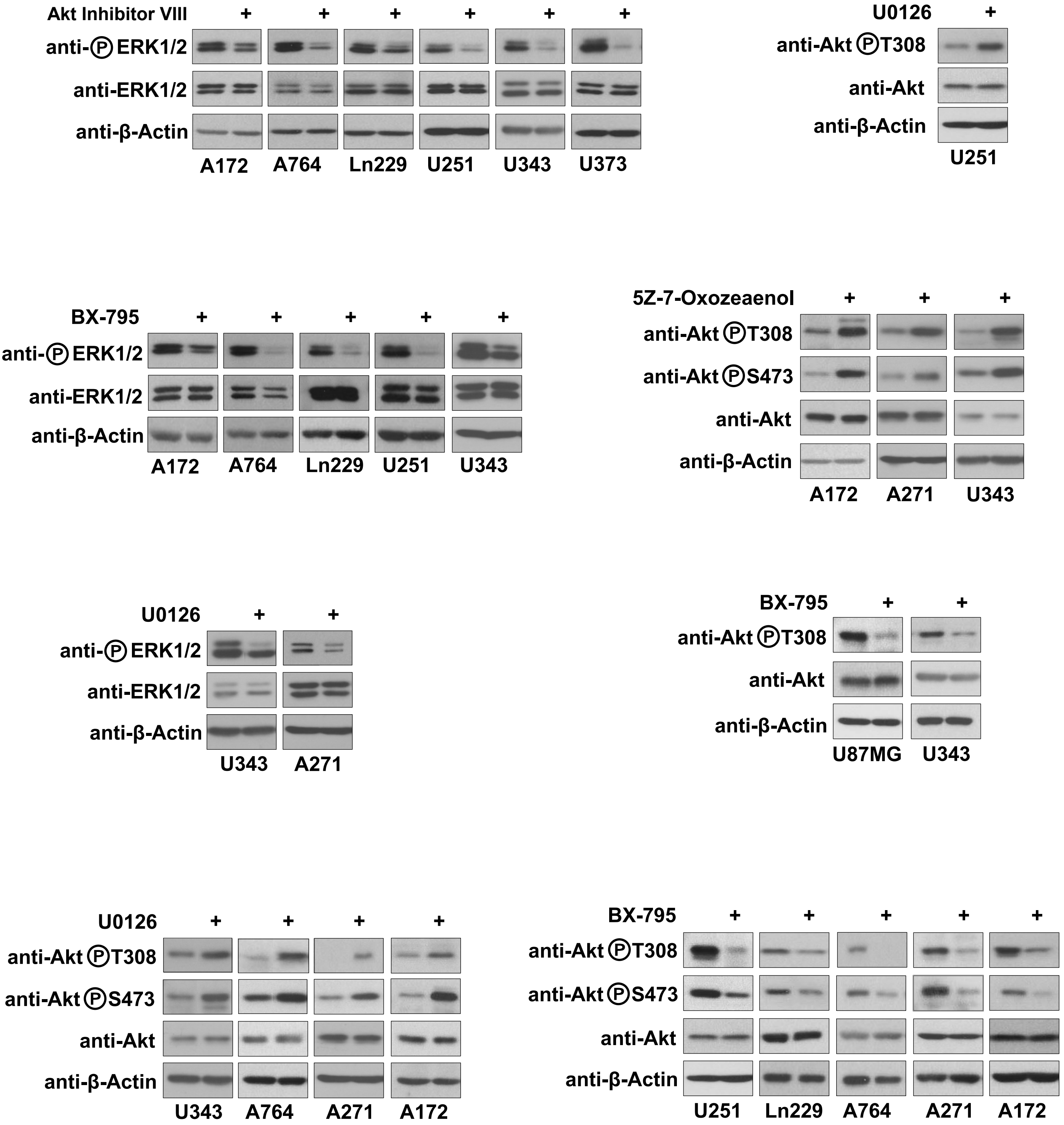

Fig. S8. Treatment of GBM cell lines with different kinase inhibitors. The indicated GBM cell lines were treated with 1 µM BX795, 5 µM Akt Inhibitor VIII, 5 µM U0126 or 1 µM 5Z-7-Oxozeaenol for 24 hs. Cell lysates were analyzed by Western Blot using antibodies against the indicated proteins or their phosphorylated forms.
