# Supplementary material for: Deregulated expression of TANK in glioblastomas triggers pro-tumorigenic ERK1/2 and AKT signaling pathways
Source: Oncogenesis. 2013 Nov 11;2(11):e79–. doi: 10.1038/oncsis.2013.42 (PMC3849693; doi:10.1038/oncsis.2013.42)
Supplement: Supplementary Table 1 [file oncsis201342x2.doc]

**Supplemental Table 1**

**Antibodies, plasmids and reagents**

**Antibodies**

| **Name** | **Species** | **Designation** | **Source** |
| --- | --- | --- | --- |
| anti-Akt | rabbit polyclonal | #9272 | Cell Signaling |
| anti-IKKε [72B587] | mouse monoclonal | ab12142 | Abcam |
| anti-p44/42 MAPK (Erk1/2) | rabbit polyclonal | #9102 | Cell Signaling |
| anti-phospho-Akt (Ser473) | rabbit polyclonal | #9271 | Cell Signaling |
| anti-phospho-Akt (Thr308) | rabbit polyclonal | #9275 | Cell Signaling |
| anti-phospho-p44/42 MAPK (Erk1/2) (Thr202/Tyr204) | rabbit polyclonal | #9101 | Cell Signaling |
| anti-TANK (D2) | mouse monoclonal | Sc-166643 | Santa Cruz |
| anti-phospho-IRF-3 (4D4G) | rabbit monoclonal | #4947 | Cell Signaling |
| anti-phospho NF-κB p65 (Ser536) (93H1) | rabbit monoclonal | #3033 | Cell Signaling |
| anti-TBK1/NAK (D1B4) | rabbit monoclonal | #3504 | Cell Signaling |
| anti-β-Actin | rabbit polyclonal | ab1801 | Abcam |
| Anti-RNA polymerase II CTD  repeat YSPTSPS (phospho S2)  antibody - ChIP Grade | rabbit polyclonal | ab5095 | Abcam |
| Secondary HRP-coupled antibodies | goat |  | Dianova |
| anti-Human HIF-1alpha | mouse monoclonal | #610959 | BD Biosciences |
| control IgG | rabbit | #2729 | Cell Signaling |

**Plasmids**

| **Plasmid** | **Origin** | **Citation** |
| --- | --- | --- |
| IFN-luciferase | human IFNβ promoter | Overby AK et al. J Virol. 2010 Sep;84(17):8470-83. |
| HA-IKKε | human IKKε | Renner F et al. Mol Cell. 2010 Feb 26;37(4):503-15. |
| HA-TBK1 | human TBK1 | Renner F et al. Mol Cell. 2010 Feb 26;37(4):503-15. |
| pSIREN-shTANK | shRNA for human TANK  5´-AGACTGAGAACTATGAGCA-3´ | Renner F et al. Mol Cell. 2010 Feb 26;37(4):503-15. |
| pSIREN-shTBK1 | shRNA for human TBK1  5´-GGAGCTACTGCAAATGTCT-3´ | this paper |
| pHCMVG |  | Addgene |
| pMDLg/pRRE |  | Addgene |
| pRSV-Rev |  | Addgene |
| pLenti-TANK | human TANK cDNA cloned into the plasmid 290-pHAGE-hEF1a CAR-PGK Puro | this paper |

**Reagents**

| **Reagent** | **Supplier** |
| --- | --- |
| BX795 | Axon Medchem |
| PD98059 | Cell Signaling |
| 1-β-D-Arabino-furanosylcytosine (AraC) | CALBIOCHEM |
| Temozolomide (TMZ) | Sigma Aldrich |
| Akt Inhibitor VIII (Isozyme-selective, Akti-1/2) | Calbiochem |
| U0126 | Cell Signaling |
| 5Z-7-oxozeaenol | Sigma Aldrich |
| Rotifect | Carl Roth |
| Propidium iodide (PI) | Sigma Aldrich |
| vinblastine | Carl Roth |

**Primers**

| **Primer name** | **Sequence (5´to 3´)** |
| --- | --- |
| ChIP_GAPDH-UP | TACTAGCGGTTTTACGGGCG |
| ChIP_GAPDH-LO | TCGAACAGGAGGAGCAGAGAGCGA |
| ChIP_hTANK_FW1 | TTTGTATGCGTGAGCGAGAG |
| ChIP_hTANK_RV1 | CGACGATGCTATGCTGACAT |
| ChIP_hTANK_FW2 | TCTTACCGCGGTTGGAATAC |
| ChIP_hTANK_RV2 | CAACTGGGGAGAGGACTGAG |
| IKKe-2-qRT-fw | GCTCAGCTCCTGGACGTGCC |
| IKKe-2-qRT-rev | TGCCCTGAGCTGGCTGGTCA |
| TBK1-qRT2-for | GGCGGAGACCCGGCTGGTAT |
| TBK1-qRT2-rev | ACATTTGCATAGCTCCTTGGCC |
| 5’UTR-TANK-r | TTCCTCTTCGTCCTGTAGCA |
| 5’UTR-TANK-f | AGGATTGTTAGAGCCTGTGGA |
| huActin-qPCR-f | TCCCTGGAGAAGAGCTACGA |
| huActin-qPCR-r | AGGAAGGAAGGCTGGAAGAG |
| TNF_FW | GTGATCGGCCCCCAGAGGGA |
| TNF_RV | ACTGGAGCTGCCCCTCAGCT |
| IL-6_FW | CCTGCACGGCATCTCAGCCC |
| IL-6_RV | TGCCAGTGCCTCTTTGTCGTC |
| Vcam_FW | ACGCTGACCCTGAGCCCTGT |
| Vcam_RV | ACGAGGCCACCACTCATCTCGA |
