# Supplementary material for: Deregulated expression of TANK in glioblastomas triggers pro-tumorigenic ERK1/2 and AKT signaling pathways
Source: Oncogenesis. 2013 Nov 11;2(11):e79–. doi: 10.1038/oncsis.2013.42 (PMC3849693; doi:10.1038/oncsis.2013.42)
Supplement: Supplementary Information [file oncsis201342x3.doc]

**Supplementary Materials and Methods**

**Cell lysis and Western blotting**

Transfected 293T cells were lysed in NP-40 buffer as described . Tumor samples were homogenised (1/10, w/v) on ice in Tris buffer (pH 7.6) containing 1 % Nonidet P-40, 5 µg/ml Pepstatin A and a protease inhibitor cocktail (Complete™, Roche). Protein concentration was determined using Bradford’s reagent. The lysates were cleared by centrifugation for 10 minutes at 13000 rpm and proteins contained in the supernatant were analyzed by Western blotting as published . The blots were exposed for various times to display signals in the dynamic range.

**Cell cycle analysis by FACS**

For the analysis of cell cycle parameters the cells were detached by incubation with Triplex Express (Gibco) and fixed with 70% (v/v) ethanol. After centrifugation of cells, the pellet was resuspended in 500 μl staining solution (RNase (200 µg/ml), Triton-X100 (0.1%(v/v) and propidium iodide (20 µg/ml)). The PI-stained DNA was detected in the FL-2 channel using a FACSCalibur device with CELLQuest software (Becton-Dickinson, Heidelberg, Germany).

**Quantitative real-time PCR**

Total RNA was isolated using the RNeasy mini kit according to the manufacturer’s instructions (Qiagen). 1 µg total RNA was reverse transcribed using SuperScript II reverse transcriptase (Invitrogen) and Oligo(dT)_12-18_ primers. Quantitative real-time PCR was performed with specific primers that are listed in supplementary Table I. All experiments were done in triplicate and data were normalized to the housekeeping gene β-actin.

**Luciferase reporter assay**

293 cells were seeded on in 6-well plates and transfected as specified in the figure legend using Rotifect (Roth). Total DNA amounts were kept equal in all transfections by adding empty expression vector. After two days cells were lysed in 200 µl NP-40 buffer and cleared by centrifugation. 30 μl of the solution as used for control Western blotting, while 10 µl of the supernatant was mixed with 10 µl of luciferase buffer and bioluminescence was immediately measured for 10 sec in a luminometer (Berthold DuoLumat LB 9501).

**References**

Renner, F., Saul, V.V., Pagenstecher, A., Wittwer, T., and Schmitz, M.L. (2011). Inducible SUMO modification of TANK alleviates its repression of TLR7 signalling. EMBO R*ep* 12, 129-135.
